# Mapping of the binding site for FcμR in human IgM-Fc

**DOI:** 10.1016/j.bbapap.2019.140266

**Published:** 2020-01

**Authors:** Rosemary A. Nyamboya, Brian J. Sutton, Rosaleen A. Calvert

**Affiliations:** Randall Centre for Cell and Molecular Biophysics, King's College London, New Hunt's House, Guy's Campus, London SE1 1UL, United Kingdom

**Keywords:** IgM antibody, Fc receptor, Immunoglobulin-like domain, Site-directed mutagenesis, Surface plasmon resonance, Fcα/μR, IgA/IgM Fc receptor, pIgR, polymeric immunoglobulin receptor, sIgFcμR, soluble extracellular immunoglobulin-like domain of FcμR, BCR, B cell receptor, IgM-Fc, dimer of Cμ2-Cμ3-Cμ4 domains, Fcμ3-4, dimer of Cμ3-Cμ4 domains, SEC-MALLS, size exclusion chromatography-multi-angle laser light scattering, SPR, surface plasmon resonance, CD, circular dichroism, RU, resonance unit in SPR, R_max_, maximum RU value, HEK, human embryonic kidney cells, IPTG, isopropyl β-d-1-thiogalactopyranoside

## Abstract

FcμR is a high-affinity receptor for the Fc portion of human IgM. It participates in B cell activation, cell survival and proliferation, but the full range of its functions remains to be elucidated. The receptor has an extracellular immunoglobulin (Ig)-like domain homologous to those in Fcα/μR and pIgR, but unlike these two other IgM receptors which also bind IgA, FcμR exhibits a binding specificity for only IgM-Fc. Previous studies have suggested that the IgM/FcμR interaction mainly involves the Cμ4 domains with possible contributions from either Cμ3 or Cμ2. To define the binding site more precisely, we generated three recombinant IgM-Fc proteins with specific mutations in the Cμ3 and Cμ4 domains, as well as a construct lacking the Cμ2 domains, and analyzed their interaction with the extracellular Ig-like domain of FcμR using surface plasmon resonance analysis. There is a binding site for FcμR in each IgM heavy chain. Neither the absence of the Cμ2 domains nor the quadruple mutant D340S/Q341G/D342S/T343S (in Cμ3 adjacent to Cμ2) affected FcμR binding, whereas double mutant K361D/D416R (in Cμ3 at the Cμ4 interface) substantially decreased binding, and a single mutation Q510R (in Cμ4) completely abolished FcμR binding. We conclude that glutamine at position 510 in Cμ4 is critical for IgM binding to FcμR. This will facilitate discrimination between the distinct effects of FcμR interactions with soluble IgM and with the IgM BCR.

## HighlightsIGHLIGHTS

• FcμR binds with similar affinity to pentameric IgM, IgM-Fc homodimer and IgM-Fc lacking the Cμ2 domains.
• Each IgM heavy chain can bind one receptor molecule.
• A single mutation in the Cμ4 domain of IgM abrogates receptor binding.

## Graphical abstract

**Figure:**
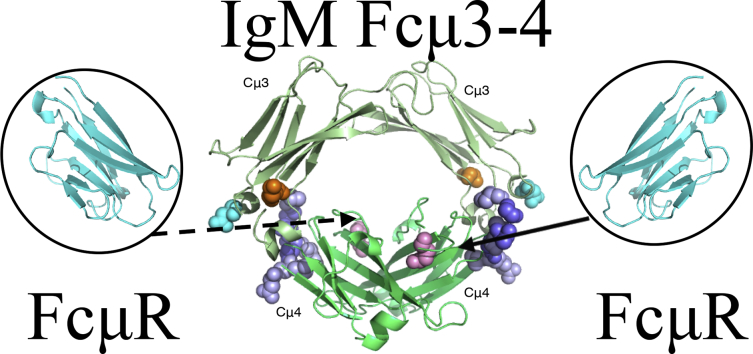

## 1 Introduction

The binding of immunoglobulins to their receptors *via* the Fc domains is key to expressing effector functions that are essential in host defense. Identification of the binding sites for these receptors on immunoglobulin Fc regions is therefore critical for understanding the molecular pathways through which they act.

Until the year 2000, the only known human IgM-Fc receptor was polymeric immunoglobulin receptor (pIgR), which binds both IgM and IgA and is expressed on basolateral surfaces of mucus epithelium and ducts of secretory glands [1]. A second receptor, designated Fcα/μR and expressed on follicular dendritic cells, macrophages and lymphocytes in humans [2,3], also binds IgM and IgA [4,5]. The recently discovered human FcμR [6] also referred to as FAIM3 or TOSO, is a high-affinity human IgM-Fc receptor expressed on B cells, T cells and a subset of NK cells [[7], [8], [9]]. The functions of FcμR are yet to be fully explored [10,11] but the receptor is thought to be involved in tonic signalling, early B cell activation and regulation of B cell-mediated T cell immunity [[12], [13], [14], [15], [16]].

FcμR is a 390-amino acid (aa) polypeptide consisting of a 17-aa signal peptide and 107-aa Ig-like domain, followed by a further 127-aa extracellular region, a 21-aa transmembrane portion that has a charged histidine residue and a 118-aa cytoplasmic tail. The receptor has no N-linked glycosylation site [7,17], however, O-linked glycosylation in the stalk region has been reported [18].

The present study focuses on the FcμR binding properties of IgM-Fc. We generated the recombinant extracellular Ig-like domain of human FcμR (sIgFcμR), IgM-Fc with and without the Cμ2 domains, and IgM-Fc with site-directed mutations, for binding analysis by surface plasmon resonance (SPR), to identify the structural determinants of IgM-Fc responsible for FcμR binding.

Previous work has shown that the Cμ3 and Cμ4 domains of polymeric IgM are involved not only in binding FcμR [6], but also the human pIgR and Fcα/μR receptors [1,19,20]. Using a panel of domain-swapped antibodies, a recent study [21] identified the Cμ4 domain as the dominant region of IgM-Fc for FcμR binding, with a minor contribution from the Cμ2 and/or the Cμ3 domains; molecular dynamics simulations of models of this interaction favoured involvement of Cμ2 residues together with Cμ4 [21]. We now report studies using site-directed mutagenesis and fragments of IgM-Fc to map more precisely the FcμR binding site and assess the contributions of the Cμ2, Cμ3 and Cμ4 domains.

## 2 Material and methods

### 2.1 Cloning and expression of sIgFcμR

The cloned receptor in Zero Blunt TOPO was kindly provided by Prof. H. Kubagawa. The coding sequence for the extracellular Ig-like domain was cloned into the plasmid expression vector pET24+ and expressed in BL21 (DE3) competent cells at 37 °C under the control of the T7 promoter. The oligonucleotides used were 5′-TGAGATCCGGCTGCTAACAAAG-3′ and 3′-TAAAACAAATTGAAATTCTTCCTCTATATGTA-5′. Cells were cultured in 1 L of ampicillin-supplemented (50 μg/mL) LB broth and grown at 37 °C with orbital shaking at 225 rpm. At an OD_600_ between 0.6 and 0.8, isopropyl β-D-1-thiogalactopyranoside (IPTG) was added to a final concentration of 1 mM to induce protein expression. The cells were grown overnight at 37 °C and pelleted by centrifugation at 4000*g* for 20 min at 4 °C. The supernatant was discarded, and the cell pellets were stored at −80 °C.

### 2.2 Purification of sIgFcμR protein

The cell pellets were thawed on ice and re-suspended in 50 mL lysis buffer (1 M NaCl, 50 mM Tris-HCL, pH 7.5 and 1 tablet of Complete EDTA-free Protease Inhibitor (Roche)). The cells were then passed through a pre-chilled cell disrupter twice, under a pressure of 1000 psi. The disrupted cells were centrifuged at 16000*g* for 15 min at 4 °C. The pellets were washed 5 times in 30 mL of 1 M NaCl, 50 mM Tris-HCL, pH 7.5 and 2% Triton-X. After the final centrifugation, the inclusion bodies were dissolved with solubilization buffer (6 M guanidine hydrochloride in 0.5 M Tris acetate buffer, pH 8.6 containing 2 mM β-mercaptoethanol), for 1 h at 37 °C. Insoluble contaminants were pelleted by centrifuging at 14000 rpm for 15 min at 4 °C, and the supernatant containing the protein collected. The sIgFcμR protein was slowly diluted 50-fold into ice-cold refolding buffer (0.1 M Tris acetate pH 8.6, 0.4 mM oxidized glutathione and 2 mM reduced glutathione), with rapid stirring at 4 °C and left to stand for 72 h. Before purification, NaCl and imidazole were added to the refold solution to a final concentration of 0.5 M and 10 mM, respectively and the pH adjusted to between 7 and 8 using 1 M acetic acid. The solution was filtered with 0.45 μM membrane filter (Millipore) before purification by nickel column chromatography. The purity of the refolded sIgFcμR was analyzed by 18% SDS-PAGE.

### 2.3 Design of IgM-Fc (Fcμ2-4) mutants

Purified serum IgM was purchased from BBI solutions. The coding sequence for His-tagged IgM-Fc (Fcμ2-4) and Fcμ3-4, lacking the tail piece residues and cloned into pcDNA5/FRT, were generously provided by Dr. Katy Doré. The plasmid containing the Fcμ2-4 sequence was the starting construct for all Fcμ2-4 mutagenesis (M1, M2 and M3). Mutations were generated using the Q5 site directed mutagenesis kit (New England Biolabs) and verified by sequencing on both DNA strands. The oligonucleotides used were, for M1 K361D mutation: 5′-CTTCCTCACCGATTCCACCAAGTTG-3′ and 3′-ATGCTGGCAAAGGATGGG-5′; for M1 D416R mutation: 5′-CATCAGCGAGCGAGACTGGAATTCCGGGGAGAGGTTCAC-3′ and 3′- CTGGCCTCACCCACGGCG-5′; for M2 Q510R mutation: 5′-GCCTGAGCCCCGAGCCCCAGGCC-3′ and 3′- ATTGGGGCGCTGGTCACATAC-5′; for M3 mutations D340S, Q341G, D342S and T343S: 5′-TCATCAGCCATCCGGGTCTTCGCC-3′ and 3’-TCCAGAGGGGACACACATGGAGGAC-5′. All primers for site directed mutagenesis were designed using NEBasechanger™ (New England Biolabs).

### 2.4 Stable expression of IgM-Fc (Fcμ2-4) mutants

All IgM proteins were stably transfected into human cells. HEK293 cells were seeded in 96-well plates to 2 × 10^5^ cells/well and cultured overnight in a 5% CO_2_ incubator at 37 °C using the transfection medium (Dulbecco's modified Eagle's medium supplemented with 10% of fetal bovine serum and 5% penicillin/streptomycin). The appropriate DNA was co-transfected with expression vector pOG44 (100 ng) using Fugene tranfection reagent (Promega) following the protocol supplied by the manufacturer. After 48 h, the cells were split into 24 well plates in transfection medium containing hygromycin B at a final concentration of 100 μg/mL. Once the cells were confluent, they were trypsinised and seeded into 1 L of Freestyle™ serum-free medium containing 5% penicillin-streptomycin and hygromycin B at a final concentration of 100 μg/mL. The cells were grown in spinners for 3–4 weeks and then harvested. Supernatants were centrifuged at 4000 *g* for 30 min and then filtered through a 0.45 μM filter (Millipore). 0.1% sodium azide was added and the supernatants were stored at 4 °C. Proteins were first purified on an AKTA Prime system (Amersham) using nickel column chromatography and then on the Gilson HPLC using a Superdex 200, 10/300 column (GE Healthcare).

### 2.5 Multi-angle laser light scattering (MALLS)

MALLS studies were performed in-line with SEC on mutant IgM protein samples to assess mono-dispersity and the molecular mass of the protein samples. The peaks corresponding to the homodimeric Fcμ2-4 mutants from SEC were run on the Superdex 200 Increase 5/150 column (GE Healthcare) using an in-line miniDAWN multi-angle light scattering detector and an Optilab DSP Interferometric Refractometer (Wyatt Technology). The data were analyzed using the ASTRA 4.9 software (Wyatt Technology).

### 2.6 Circular dichroism (CD) analysis

Far-UV (190-280) CD spectra were acquired on a Chirascan Plus spectropolarimeter (Applied Photophysics Ltd) flushed continuously with pure evaporated nitrogen throughout the experiments. Measurements were recorded in a 0.5 mm strain-free rectangular cell using a 2 nm spectral bandwidth, 1 nm step-size and 1 s measurement time-per-point. The concentrations of the protein samples used ranged between 0.1 mg/mL - 0.4 mg/mL and buffer used was 10 mM HEPES and 150 mM NaCl at pH 7.4. All spectra were acquired at 25 °C and buffer baseline corrected before analysis.

### 2.7 Surface plasmon resonance (SPR) analysis

All SPR assays were performed on a Biacore T200 instrument (Biacore, Uppsala, Sweden) at 25 °C in running buffer (0.01 M HEPES, 0.15 M NaCl and 0.005% Surfactant P20, pH 7.4). Serum IgM, IgM-Fc, Fcμ3-4 and IgM-Fc (Fcμ2-4) mutants were immobilized on Biacore CM5 chips in 10 mM sodium acetate, pH 4.5, using standard amine coupling chemistry. The analyte was sIgFcμR in concentrations ranging from 0.25 μM to 12 μM. In each experiment, the binding curves were corrected by subtracting the signal obtained from the control flow cell. Non-specific binding was <30%. Steady state binding curves were analyzed using Biacore T200 evaluation software. The errors in the K_D_ values represent the fitting of the 1:1 binding model to the experimental data.

To investigate the stoichiometry of sIgFcμR binding to IgM or its homodimeric fragments, the following equation was used to calculate the activity of the immobilized IgM or fragments (which can be up to 100%, depending on whether any is damaged or misoriented by immobilization):

% Activity = 100 × (Mol. Wt. of IgM or homodimeric fragment × R_max_ of sIgFcμR bound)/ (Mol. Wt. of sIgFcμR × RU of IgM or homodimeric fragment immobilized).

## 3 Results

**Fig. 1:**
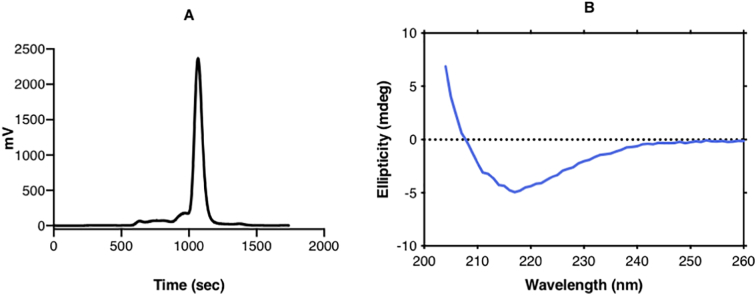
Preparation and characterization of FcμR Ig binding domain (sIgFcμR). sIgFcμR protein was purified by SEC (panel A) and analyzed using far-UV CD spectroscopy at 0.1 mg/mL (panel B).

**Fig. 2:**
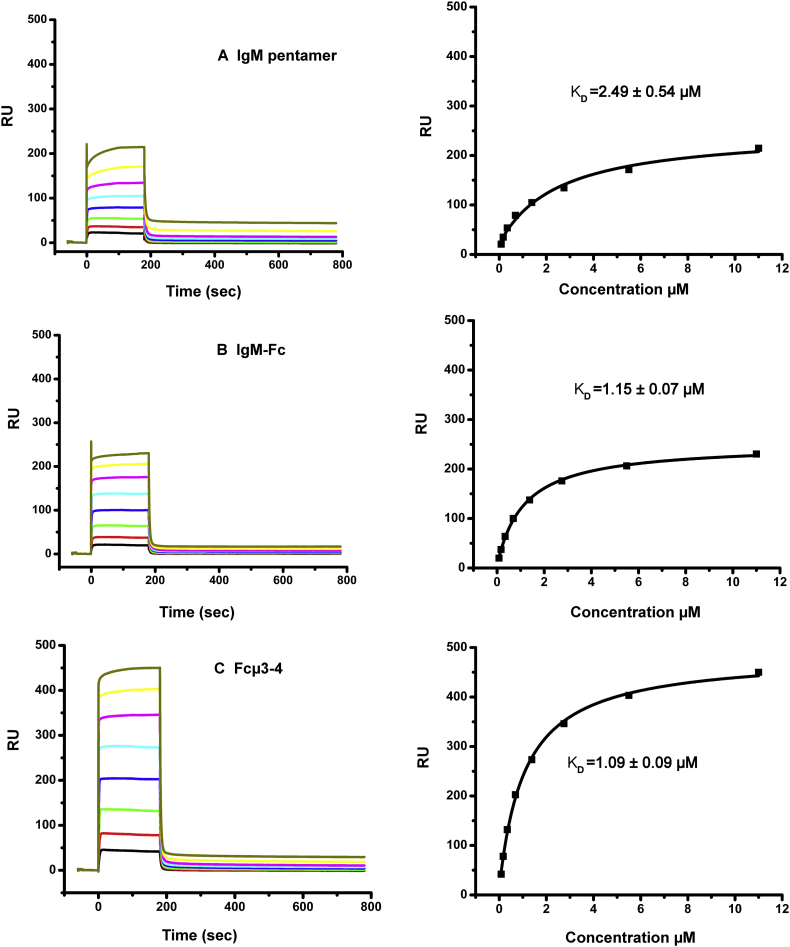
SPR analysis of sIgFcμR interaction with IgM, IgM-Fc and Fcμ3-4. Whole pentameric IgM, (panel A), IgM-Fc (panel B) and Fcμ3–4 (panel C), were immobilized on a CM5 sensor chip and binding of sIgFcμR was determined as described in Material and Methods. Experiments were conducted using at least six sIgFcμR concentrations. The KD values were generated from the steady state binding curves using Biacore T200 evaluation software.

The extracellular Ig-like domain of human FcμR (sIgFcμR) was expressed as described in Material and Methods. After affinity purification, SEC was conducted to ensure the purity of the protein (Fig. 1A). Fractions from the main peak were collected and further checked by analytical SEC before SPR analysis; the fraction used co-migrated in SEC with cytochrome *c* (molecular weight 12.4 kDa). The far-UV CD spectrum showed a characteristic β-sheet signature of a folded polypeptide with a broad minimum near 218 nm (Fig. 1B). SPR analysis of sIgFcμR binding to IgM, IgM-Fc (also termed Fcμ2-4, consisting of two each of the Cμ2, Cμ3 and Cμ4 domains) and Fcμ3-4 (lacking the Cμ2 domains), yielded K_D_ values of 2.49 ± 0.54 μM, 1.15 ± 0.07 μM and 1.09 ± 0.09 μM respectively (Fig. 2). This showed that neither pentamerisation, presence of the Fabs nor the Cμ2 domains substantially affected binding of sIgFcμR.

SPR has limitaitons as a method to determine the absolute stoichiometry between, in this case, an antibody or fragment and its receptor, due to inevitable loss of activity caused by chemical coupling of the antibody or fragment to the matrix. However, since the initial baseline signal in RU is proportional to the mass of antibody or fragment immobilizsed, and the additional signal in RU is proportional to the mass of receptor bound, it is possible to calculate the theoretical maximum expected signal assuimg a particular stoichiometry of binding. For each of the proteins, IgM, IgM-Fc and Fcμ3-4, the ratio of R_max_ (the extrapolated maximum SPR signal for sIgFcμR binding in resonance units, RU) to the number of RU immobilized on the sensor chip, taking into account the relative molecular weights (900 kDa, 76 kD and 51 kDa respectively), indicated that each IgM heavy chain has a binding site for sIgFcμR. The number of RU immobilized for IgM, IgM-Fc and Fcμ3-4 was 1959, 876 and 1343 respectively, and the sIgFcμR R_max_ values were 229, 253 and 472. If the stoichiometry of binding is assumed to be one sIgFcμR to one IgM-Fc homodimer, then the apparent binding activities are 165%, 171% and 139% respectively (see equation in Material and Methods); since no immobilized protein can have an activity of >100% (and it is commonly much less), this indicates that each heavy chain must be able to bind one sIgFcμR molecule (*i.e.* 2:1 sIgFcμR:IgM-Fc homodimer).

Site-directed mutagenesis was applied to study IgM-Fc binding to sIgFcμR using SPR. While there is currently no crystal structure for human IgM-Fc or any of its domains, assumptions about their structural features can be made based on the individual domain structures of mouse IgM-Fc [22] and the known structures of other immunoglobulin class Fc regions and their complexes. IgM and IgE are the only two human immunoglobulin classes with an extra pair of domains, Cμ2 and Cε2 respectively, in place of a flexible hinge region. Additionally, the human Cμ2-4 domains show 29% identity and 48% similarity to the corresponding region of human IgE at the amino acid level. Consequently, the human IgE-Fc/sFcεRIα and IgE-Fc/sCD23 crystal structures [23,24], as well as other immunoglobulin-Fc/receptor structures [25,26], were used to predict likely binding sites for FcμR in IgM-Fc. Furthermore, residues in IgA Cα3 have been implicated in pIgR binding [[27], [28], [29]], and there is an homologous loop in IgM Cμ4. Thus IgM-Fc residues structurally homologous to those involved in IgE-Fc/FcεRIα, IgE-Fc/CD23, IgA-Fc/FcαR and IgA-Fc/pIgR interactions were targeted with mutations. A striking similarity between the nature and location of the binding sites in IgG and IgE for their Fc receptors has been observed [26,30], and thus the proposition that the IgM-Fc/FcμR binding site might be structurally homologous to one of these interactions is not unreasonable.

**Fig. 3:**
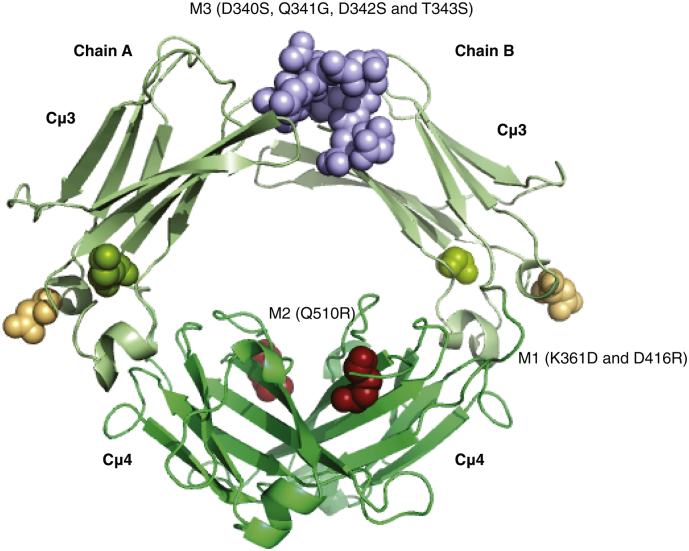
Schematic showing the mutation sites in the Cμ3 and Cμ4 domains. Homology model of human Fcμ3–4 showing the locations of mutated IgM residues in space-filling representation: K361D (green)/D416R (straw), Q510R (red) and the adjacent residues 340–343, DQDT to SGSS (purple). The IgM-Fc structure was predicted by the Swiss Model Server [31] using the structure of human IgE-Fc (PDB ID: 2WQR) as a template.

Fig. 3 shows the locations of the mutations introduced into the Cμ3 and Cμ4 domains of IgM-Fc. Three mutant human IgM-Fc (Fcμ2-4) constructs were designed. In mutant M1, aspartic acid and arginine residues were introduced at positions 361 and 416 respectively (K361D/D416R). These residues are analogous to those critically involved in the IgE-Fc/CD23 and IgA-Fc/FcαR interactions, respectively. In mutant M2, a glutamine residue at position 510 was replaced by arginine (Q510R) at a site corresponding to the pIgR interaction with IgA-Fc. Mutant M3 involved residues 340–343 in the Cμ3 domain (D340S/Q341G/D342S/T343S), which correspond to critical components of the IgE-Fc/FcεRIα and IgG-Fc/FcγR interactions.

**Fig. 4:**
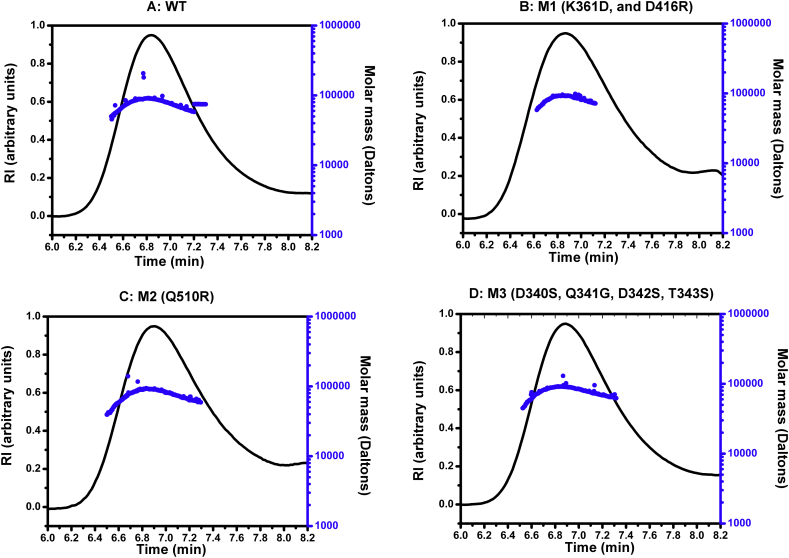
Characterization of IgM-Fc wild-type and mutants, M1, M2 and M3. SEC-MALLS analysis of IgM-Fc wild-type (panel A) and mutants M1 (panel B), M2 (panel C) and M3 (panel D). The refractive index detector signal (black), and calculated molar mass (blue), are plotted as a function of the column elution time.

The IgM-Fc mutants were secreted from human embryonic kidney (HEK) cells. After purification by affinity chromatography followed by SEC (data not shown), the proteins were characterized by SEC-MALLS and far-UV CD. Similarly, non-mutated IgM-Fc (wild-type) was prepared to act as a positive control in the experiments. For all four preparations, SEC-MALLS analysis revealed single peaks with a weight-averaged molar mass between 80 and 84 kDa, within experimental error of one another and of IgM-Fc homodimer, proving that the samples were not aggregated (Fig. 4).

**Fig. 5:**
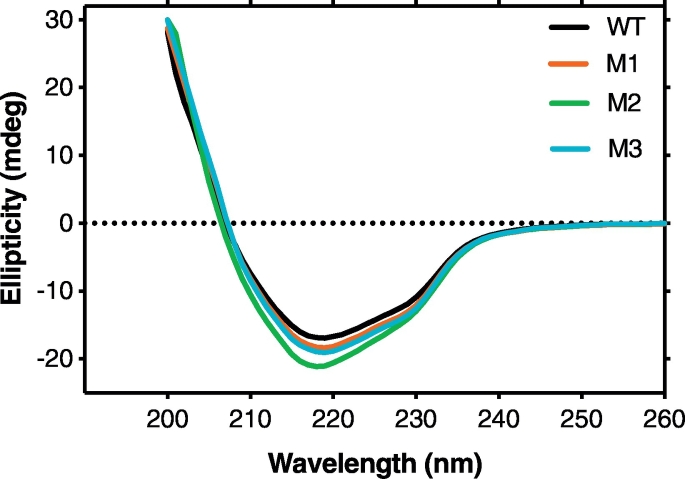
Characterization of IgM-Fc wild-type (WT) and mutants, M1, M2 and M3. Far-UV CD spectra for all four proteins are characteristic of β-sheet structure (all scaled to a concentration of 0.4 mg/mL).

The far-UV CD profiles of all the three IgM-Fc mutants compared well with that of wild-type, suggesting that the native states of the mutants were not perturbed by the respective mutations and that all were properly folded (Fig. 5).

**Fig. 6:**
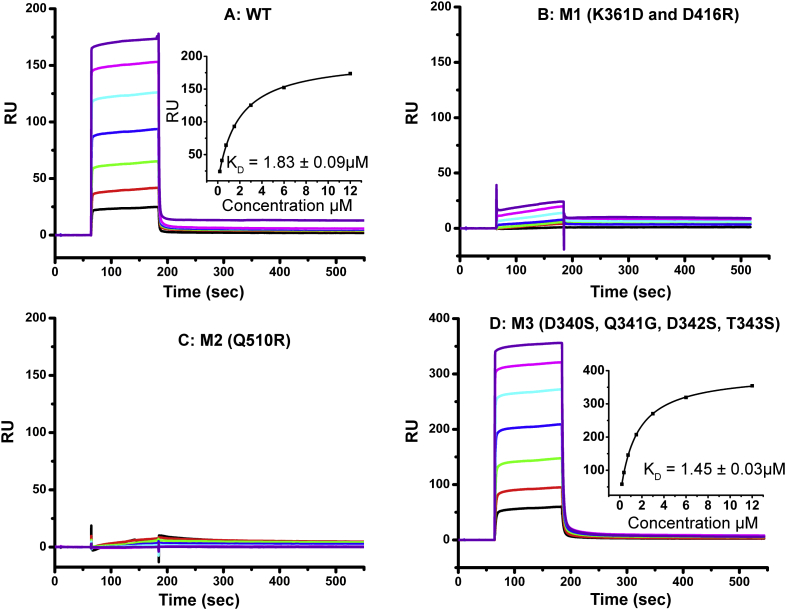
SPR analysis of sIgFcμR interaction with IgM-Fc wild-type and mutants, M1, M2 and M3. IgM-Fc wild-type (panel A) and mutants, M1 (panel A), M2 (panel C), and M3 (panel D) were immobilized on a CM5 sensor chip and binding of sIgFcμR was determined as described in Material and Methods. Experiments were conducted using at least six sIgFcμR concentrations, with replicates. The KD values were generated from the steady state binding curves using Biacore T200 evaluation software.

SPR analysis was used to assess the binding of sIgFcμR to immobilized wild-type, single- (Q510R), double- (K361D/D416R) and quadruple- (D340S/Q341G/D342S/T343S) mutant IgM-Fc. The sensorgrams for the four IgM-Fc species and the resulting K_D_ values for wild-type and the quadruple mutant are shown in Fig. 6.

The mutation of residues 340–343 in Cμ3 generated an IgM-Fc mutant (M3) with sIgFcμR binding parameters (K_D_ = 1.45 ± 0.03 μM) almost indistinguishable within experimental error from the wild-type (K_D_ = 1.83 ± 0.09 μM). This shows that residues D340, Q341, D342 and T343, which correspond to key elements of the IgE-Fc/FcεRIα and homologous IgG-Fc/FcγR interactions, play no role in the binding of IgM-Fc to FcμR. The double mutation of residues 361 and 416 in Cμ3 (M1) substantially reduced sIgFcμR binding; the location of K361 and D416 at the Cμ3/Cμ4 interface corresponds to that of the CD23 and FcαR binding sites in IgE and IgA respectively. (The low signal does not permit calculation of a reliable K_D_ value). Further investigation with single mutations would be required to determine the contribution of each. Finally, mutation of residue 510 in Cμ4 (M2) showed no binding of sIgFcμR at all concentrations tested, indicating that Q510 plays a critical role in the IgM-Fc/FcμR interaction.

## 4 Discussion

When the receptor FcμR was first identified, the binding region in IgM was shown to lie (principally) within the Cμ3 and Cμ4 domains since an Fcμ_5_ fragment, consisting largely of these domains, inhibited the interaction [6,17]. A later study with “domain-swapped” antibody Fc regions confirmed the involvement of the Cμ4 domain and implicated a possible contribution from either Cμ2 or Cμ3 [21]. In order to locate more precisely the binding site for FcμR in IgM-Fc, we produced for the first time the recombinant extracellular Ig-like domain of FcμR (sIgFcμR) in *E. coli*, and studied its binding to IgM, IgM-Fc, a series of IgM-Fc mutants and a sub-fragment of IgM-Fc lacking the Cμ2 domains.

The binding of purified sIgFcμR to whole serum IgM (largely pentameric), IgM-Fc (Fcμ2-4), and the Fc sub-fragment Fcμ3-4, was investigated by SPR (Fig. 2). It is clear that sIgFcμR interacts with both whole IgM and the two Fc fragments with similar binding affinities, indicating that the Cμ2 domains do not contribute to FcμR binding. These results also show that unlike pIgR and Fcα/μR [19,20], FcμR can bind to both homodimeric and polymeric IgM. Assuming a 1:1 stoichiometry of each heavy chain to sIgFcμR, a binding affinity of K_D_ ~ 1–2.5 μΜ was determined by SPR. This is ~ 100 fold weaker than cell surface FcμR binding to pentameric serum IgM, which is reported as K_D_ ~ 10 nM [6]; the difference is presumably due to the very high effective concentration of FcμR on the cell surface and the possibility of simultaneously engaging more than one receptor domain (*i.e.* an avidity effect).

In order to determine the FcμR binding site on IgM, we generated three IgM-Fc mutants, M1, M2 and M3 (Fig. 3) and studied their interaction with the receptor sIgFcμR by SPR (Fig. 6). Mutations in Cμ3 (M3, Fig. 3) corresponding to the location of the high-affinity receptor FcεRI in IgE-Fc, had no effect upon sIgFcμR binding. In contrast, mutation Q510R in Cμ4 (M2, Fig. 3) abrogated receptor binding completely. A glutamine residue lies within the structurally homologous loop in the Cα3 domain of IgA-Fc, which has been implicated in the IgA-Fc/pIgR interaction [27]. A double mutation (K361D/D416R) in Cμ3 (M1, Fig. 3), adjacent to Cμ4 in the region to which CD23 binds in IgE, and FcαR in IgA-Fc, also substantially reduces sIgFcμR binding. These results establish that residue Q510, located in an exposed loop region of Cμ4, is a key component of the binding site for FcμR that may also encompass an adjacent part of Cμ3.

**Fig. 7:**
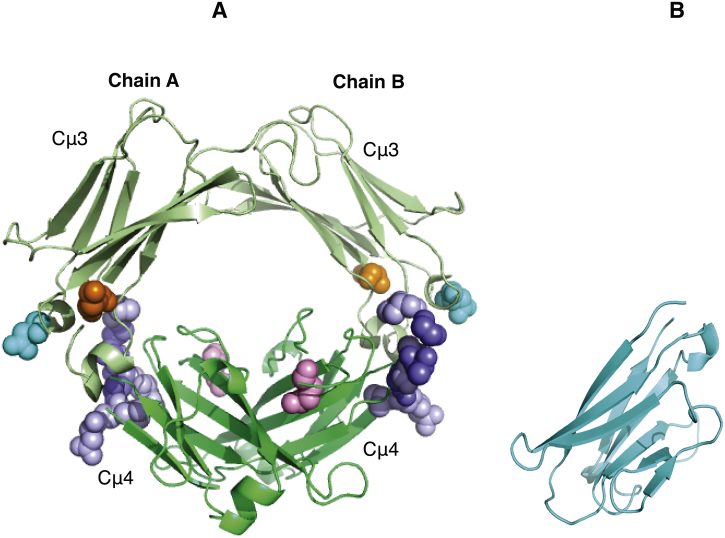
Models of Fcμ3-4 and sIgFcμR showing the proposed receptor binding site. Panel A: model of Fcμ3-4 showing residues suggested to play a role in the IgM-Fc/FcμR interaction. The hydrophobic and polar residues within the interface in the model proposed by Lloyd et al. [21] are coloured in blue and light purple, respectively. The residues K361D, D416R and Q510R reported in our study are coloured in orange, cyan and pink, respectively. Panel B: model of sIgFcμR. The models of Fcμ3-4 and sIgFcμR were predicted using the Swiss Model server [31]. The template for Fcμ3-4 was IgE-Fc (PDB ID: 2WQR) and for sIgFcμR the template was camelid VHH fragment (PDB ID: 5JMR).

Lloyd et al. proposed a model of the interaction between IgM-Fc and FcμR, based upon molecular dynamics simulations, that involved several residues from Cμ4, but not Q510, together with a contribution from Cμ2 [21]. Their experimental data implicated involvement of Cμ4 with minor contributions from either Cμ2 or Cμ3. Our data suggest that it is Cμ3, but not Cμ2, that may be involved together with Cμ4. Q510 is however adjacent to Cμ4 residues in the model of Lloyd et al., and taken together they may define an extended binding region (Fig. 7A) that could be encompassed by the (modelled) Ig-like domain of FcμR (Fig. 7B).

In cryoEM structures and models of pentameric and hexameric IgM [[32], [33], [34]], the Q510 loop and adjacent Cμ4 residues are accessible. Residues 361 and 416 however, lie at the interface between subunits in these polymeric structures, although they would presumably be exposed in membrane IgM expressed on B cells as part of the B cell receptor (BCR) for antigen. The FcμR binding site very likely overlaps with part of that of pIgR, by analogy to the location identified in IgA Cα3 [[27], [28], [29]] and the fact that Cμ4 is involved in binding to IgM [19]; furthermore, overlap with the Fcα/μR binding site is also likely since residues identified as involved in Fcα/μR binding to IgA are located in adjacent loop regions [20].

The functional significance of this clustering of IgM receptor binding sites in Cμ4 is unclear, although some different cell types which express these receptors do co-localise (*e.g.* FDCs and B cells in germinal centres or epithelial cells and B cells) and could lead to competition for IgM. However, the Cμ4 domain has also been identified as the binding site for the erythrocyte membrane protein pfEMP1 expressed by cells infected with *Plasmodium falciparum* [35], and interference with FcμR binding has been proposed as a possible means by which this malarial parasite evades the host immune system [36].

## 5 Conclusions

There have been contradictory findings in relation to the function of FcμR [10]. The identification of a critical amino-acid residue substitution in Cμ4 (Q510R) now permits the preparation of pentameric IgM which cannot bind FcμR; this could be used to investigate B cell reactions in which FcμR can only interact with the IgM BCR. Indeed, if this mutation could be effected in the IgM BCR, then the proposed interaction with FcμR and its role in B cell survival, IgM BCR expression and tonic signalling [13,15] could be tested directly.

## Author contributions

R.A.C. and B.J.S. designed the research. R.A.C. and R.A.N. performed and analyzed the experiments. All authors contributed to writing the paper.

## Credit author statement

Rosemary Nyamboya: Formal Analysis, Investigation, Visualization, Writing-Original Draft, Writing-Review and Editing.

Brian Sutton: Conceptualization, Funding, Project Administration, Writing-Review and Editing.

Rosaleen Calvert: Conceptualization, Formal Analysis, Investigation, Project Administration, Supervision, Validation, Visualization, Writing-Review and Editing.

## Declaration of Competing Interest

The authors declare that they have no conflicts of interest with the contents of this article.
